# Geodemographics profiling of influenza A and B virus infections in community neighborhoods in Japan

**DOI:** 10.1186/1471-2334-11-36

**Published:** 2011-02-02

**Authors:** Yoshinari Kimura, Reiko Saito, Yoshiki Tsujimoto, Yasuhiko Ono, Tomoki Nakaya, Yugo Shobugawa, Asami Sasaki, Taeko Oguma, Hiroshi Suzuki

**Affiliations:** 1 Division of Public Health, Department of Infectious Disease Control and International Medicine, Niigata University Graduate School of Medical and Dental Sciences, Niigata, Japan; 2 Department of Geography, Graduate School of Literature and Human Sciences, Osaka City University, Osaka, Japan; 3 Isahaya City Medical Association, Nagasaki, Japan; 4 Department of Geography, Ritsumeikan University, Kyoto, Japan; 5 Department of Nursing, Niigata Seiryo University, Niigata, Japan

## Abstract

**Background:**

The spread of influenza viruses in a community are influenced by several factors, but no reports have focused on the relationship between the incidence of influenza and characteristics of small neighborhoods in a community. We aimed to clarify the relationship between the incidence of influenza and neighborhood characteristics using GIS and identified the type of small areas where influenza occurs frequently or infrequently.

**Methods:**

Of the 19,077 registered influenza cases, we analyzed 11,437 influenza A and 5,193 influenza B cases that were diagnosed by the rapid antigen test in 66-86 medical facilities in Isahaya City, Japan, from 2004 to 2008. We used the commercial geodemographics dataset, Mosaic Japan to categorize and classify each neighborhood. Furthermore, we calculated the index value of influenza in crude and age adjusted rates to evaluate the incidence of influenza by Mosaic segmentation. Additional age structure analysis was performed to geodemographics segmentation to explore the relationship between influenza and family structure.

**Results:**

The observed number of influenza A and B patients in the neighborhoods where young couples with small children lived was approximately 10-40% higher than the expected number (p < 0.01) during all seasons. On the contrary, the number of patients in the neighborhoods of the aging society in a rural area was 20-50% lower than the expected number (p < 0.01) during all seasons. This tendency was consistent after age adjustment except in the case of influenza B, which lost significance in higher incidence areas, but the overall results indicated high transmission of influenza in areas where young families with children lived.

**Conclusions:**

Our analysis indicated that the incidence of influenza A and B in neighborhood groups is related to the family structure, especially the presence of children in households. Simple statistical analysis of geodemographics data is an effective method to understand the differences in the incidence of influenza among neighborhood groups, and it provides a valuable basis for community strategies to control influenza.

## Background

Influenza is a highly contagious acute respiratory disease that causes periodic seasonal epidemics and global pandemics, and shows marked seasonality in many countries [1,2]. The spread of influenza viruses in a community is influenced not only by the type of virus [3,4], but also by factors such as age [5,6], immunological conditions of person [7,8], climate [9,10], indoor crowding [11,12], school activity [13-15], and social contacts [16-22].

Recently, the geographical spread of seasonal influenza was investigated with the aid of geographic information systems (GIS) [23-27]. We found that the spread of influenza in Japan showed a particular pattern every year from western-central Japan to northeast Japan [25].

Geodemographics is widely defined as "analyses of people by where they live", and is constructed by linking classified neighborhoods [28] and some indices of interest such as economy, health, crime, or education. One of the commonly used commercial geodemographics tools is Mosaic, which was originally developed in the UK. The Japanese version of this product classifies 0.2 million census districts into 11 Groups and 50 Types by clustering socio-economic and demographic variables.

Until now, no reports have focused on the relationship between the incidence of influenza and characteristics of small neighborhoods in a community. We started to map the incidence of influenza at the census enumeration district level in a local city, Isahaya City in Nagasaki Prefecture, Japan using an influenza patient registration program conducted by the Isahaya Medical Association since 2004. Nearly all pediatric and internal medicine outpatient clinics joined the project.

By combining influenza mapping and commercial geodemographics segmentation (Mosaic Japan) at the small enumeration district level, we aimed to profile neighborhoods where influenza frequently or infrequently occurs. Using these results, we speculated the socio-demographic factors affecting the transmission of influenza in a community.

## Methods

### Study Area

The study area comprised the Isahaya and Tarami areas, affiliated to Isahaya City in Nagasaki Prefecture, located in the southwestern part of Japan. The region had a population of approximately 113,000 in the 2005 census. Its total area of approximately 183 km^2 ^is subdivided into 105 small areas (census enumeration districts).

### Influenza and Demographic Data

The Isahaya City Medical Association in Isahaya City, Nagasaki Prefecture began an influenza patient registration program during the 2003/04 influenza season. Subsequently, the Department of Public Health, Graduate School of Medical and Dental Sciences, Niigata University, Niigata City, Niigata Prefecture, joined the project to visualize patient locations using GIS mapping. Under this program, information on influenza patients who visited cooperating medical facilities was collected. The number of facilities that participated during the four influenza seasons from 2004 to 2008 was 66, 86, 81, and 74, respectively. Of note, all pediatric and internal medicine outpatient medical facilities in the area (53, 55, 57, and 58 for the four seasons from 2004 to 2008, respectively) were included. Other specialists such as otorhinolaryngologists also cooperated, and thus, the number of medical facilities that participated was higher than that of the pediatric and internal medicine facilities.

Patients who visited the medical facilities with influenza-like-illness, such as having a sudden fever (> 38°C) and sore throat, cough, or chills were eligible for the study.

Next, their nasopharyngeal swabs or aspirates were examined using rapid antigen test kits for the diagnosis of influenza A or B, a common clinical practice in Japan. Some patients were clinically diagnosed as having influenza without rapid antigen testing. After obtaining informed consent, we collected the following information of the patients: sex, age, onset day, result of rapid test (Type A or B or clinical diagnosis of influenza-like illness), and census enumeration district level residential address. However, the refusal cases, influenza rapid antigen test negative cases, and names of medical facilities that the patients visited were not recorded in this study.

Since our study design was not experimental and comprised no interventions shared only with medical and local government personnel in the city, a formal ethical review of this study was not sought before conducting the survey. However, upon the start of the collaborative study, the ethical committee in Isahaya Medical Association reviewed the study proposal and approved that the accumulated data could be analyzed in Niigata University and Osaka City University for publication, under the condition that anonymity of patient's data was guaranteed.

We obtained corresponding population data for 5-year age groups by enumeration district from the 2005 national census which was published by the Statistics Bureau of Japan. These data were used to analyze the incidence of influenza A and B and age structures in the entire study area and each neighborhood category.

We calculated the population-based influenza incidence (II_*k*_) for each age group *k*, type of influenza virus (A or B), and season using the number of influenza cases in age group *k *(C_*k*_) and the number of people in age group *k *in 2005 (N_*k*_), as follows:

$${\text{II}}_{k}=\frac{{\text{C}}_{k}}{{\text{N}}_{k}}\times 100$$

Because the sensitivity and specificity of the rapid test have been shown to be good indicators of influenza infection in Japan (sensitivity 82.7-98%, specificity 93.9-100%) [29], we analyzed only influenza A or B positive patients by the rapid tests and excluded those diagnosed clinically.

### Mapping of Influenza Incidence

To visualize the spread of influenza infections in the community, we geocoded the residential locations of patients at the census enumeration district level and made a map to show incidence by districts using GIS software, ArcGIS Desktop 9.2 (Environmental Systems Research Institute. Redland, CA).

### Geodemographics Data

Geodemographics data is used to identify the type of residents living in a neighborhood. Mosaic Japan is a commercial geodemographics segmentation dataset developed by Acton Wins Co., Ltd (Osaka, Japan) in partnership with Acton International, Ltd. (Lincoln, NE) and Experian Co., Ltd. (Nottingham, UK). Mosaic Japan classifies all of Japanese 211,000 census enumeration districts into 11 major neighborhood groups, and subdivides them into 50 different types. These 11 groups and 50 types are called the Mosaic Groups and Mosaic Types, respectively. These groups and types are designed to reflect the dominant classes in the area and cover the socio-cultural diversity of all neighborhoods in Japan. Table 1 gives a description of each Mosaic Group in the Mosaic Japan dataset (see URL: http://www.awkk.co.jp/mosaic/ for details of the 50 Mosaic Types). Nevertheless, precise information regarding the composition of various demographic and socio-economic indices and their proportion in each Mosaic Group and Type has not been released by the manufacturer.

**Table 1:** Neighborhood group profile in Japan by Mosaic Japan Group

Group	Group Description	Neighborhood Profile
A	Metropolitan Careerists	Metropolitan Careerists tend to be under forty and earn a very high income. Many of them fall into the top tax bracket.
B	Graduate Newcomers	Young families with children living in modern apartments in the new residential areas of small cities and the suburbs of large cities.
C	Campus Lifestyles	Campus Lifestyles are found in relatively small towns, where college or graduate students live. These areas are sometimes research centers.
D	Older Communities	Typical inner areas of small or middle sized cities, where many old people over sixty have lived for more than twenty years.
E	Middle Japan	A balanced mixture of different types of people, including young families and middle-age families, living in typical Japanese towns.
F	Corporate Success Story	Employees of well-established corporations, who have worked their way up the ranks and obtained a certain level of social status.
G	Burdened Optimists	Families in their 30s and 40s that have recently moved into detached houses and apartments in new residential areas to raise their children.
H	Social Housing Tenants	Low wage earners living in large cities in middle to large apartment blocks of social housing developed by local authorities.
I	Blue Collar Owners	Small industrial towns whose main business is in the manufacturing industry and many residents are skilled workers in local factories.
J	Rural Fringe	Periphery of cities or areas close to provincial cities, where many residents work in the agricultural.
K	Deeply Rural	People living in agricultural villages, which are remote from urban areas and sometimes totally isolated from the outside world.

Source: Mosaic Japan website; http://www.awkk.co.jp/mosaic/

### Influenza Incidence Analysis by Mosaic Group and Type

We correlated the number of influenza patients and 5-year age group populations to the Mosaic Japan dataset using the Join Table function in ArcGIS Desktop 9.2, and then the total number of cases from each census enumeration district was aggregated by Mosaic Group and Type.

Next, we calculated the expected number of cases (EC_*i*_) under both non-age-adjusted and age-adjusted conditions by Mosaic Group and Type for each virus type and season using the incidence of influenza as follows:

$${\text{EC}}_{i}=\text{II}\times \frac{{\text{N}}_{i}}{100}\;or{\text{ II}}_{k}\times \frac{{\text{N}}_{i}}{100}$$

where II is the incidence of influenza in the study area for each virus type and season (non-age-adjusted condition) and II_*k *_is the population-based influenza incidence for each age group *k *for each type and season (age-adjusted condition).

Finally, we computed the index value of influenza incidence (IVII_*i*_) by Mosaic Group and Type for each virus type and season as follows:

$${\text{IVII}}_{i}=\frac{{\text{C}}_{i}}{{\text{EC}}_{i}}\times 100$$

where C_*i *_is the number of influenza cases in the *i*th category.

When the value of IVII_*i *_in a neighborhood group (Mosaic Group or Type) is 100, the rate of incidence of influenza in this group is the same as that expected, and an IVII_*i *_value of 200 indicates that the observed number of cases is 100% higher than that expected in the study area.

### Statistical Analysis

Differences between the observed and expected number of patients were assessed by the Pearson chi-square test, and p < 0.05 was considered statistically significant. The chi-square statistics was used to determine the degree to which the reported number of patients differed from the expected number in individual neighborhood groups. We calculated these indicators for each influenza virus type and season by the Mosaic Group and Type.

We excluded 3 census enumeration districts from the statistical analysis because these districts were sparsely populated (5 people or fewer). Because of the small number of cases, we did not calculate indicators for influenza B in the 2005/06 and 2007/08 seasons. All calculations were performed with Microsoft Excel 2003 (Microsoft Corp., Redmond, WA).

### Age Structure Analysis by Mosaic Group and Type

Because the precise age structure information for each Mosaic segmentation was not disclosed by the manufacturer, we performed additional analyses to elucidate the relationship between age structure of each cluster and the incidence of influenza.

We calculated the index value of each 5-year age group (IVAG_*ik*_) for Mosaic Group and Type in the study area. The IVAG_*ik *_for a specific age group *k *in each Mosaic Group and Type was calculated using the following equations.

First, the proportion of age group *k *(PAG_*k*_) in the study area was calculated as follows:

$${\text{PAG}}_{k}=\frac{{\text{N}}_{k}}{\text{N}}\times 100$$

where N_*k *_and N are the number of people of age group *k *and the total population size in the study area, respectively.

Second, the expected populations of age group *k *(EP_*ik*_) were calculated by Mosaic Group and Type as follows:

$${\text{EP}}_{ik}={\text{PAG}}_{k}\times \frac{{\text{N}}_{i}}{100}$$

where N_*i *_is the total number of people in the *i*th category of the Mosaic Group or Type.

Third, IVAG_*ik *_for each Mosaic Group and Type was calculated using EP_*ik *_as follows:

$${\text{IVAG}}_{ik}=\frac{{\text{N}}_{ik}}{{\text{EP}}_{ik}}\times 100$$

where N_*ik *_is the number of people of age group *k *in the *i*th category.

When the value of IVAG_*ik *_in a Mosaic Group or Type is 100, the rate of age group *k *is equal to the expected for this group in the study area. When the value is more or less than 100, the rate of the 5-year age group is higher or lower than that expected, and a value of 200 indicates that the rate is twice of that expected in the study area.

### Population density by Mosaic Group and Type

We calculated the population density (number of people per square kilometer) by Mosaic Group and Type by dividing the aggregated number of population from the national 2005 census and the aggregated area of enumeration districts.

## Results

### Influenza Epidemics from the 2004/05 to 2007/08 Seasons in the Study Area

In total, 19,077 influenza-like-illness cases were registered during the four influenza seasons from 2004/05 to 2007/08 in the study area. Overall, we analyzed 16,465 cases (86.3%), comprising 11,319 patients (59.3%) with influenza A and 5,146 (27.0%) with influenza B. As a definition, 2,477 patients (12.8%) who were clinically diagnosed with influenza were excluded from analysis. According to the Isahaya City Medical Association, the refusal rate for participation was very small. Furthermore, the number of cases that were negative as shown by the influenza rapid antigen test kit was unknown.

**Figure 1:**
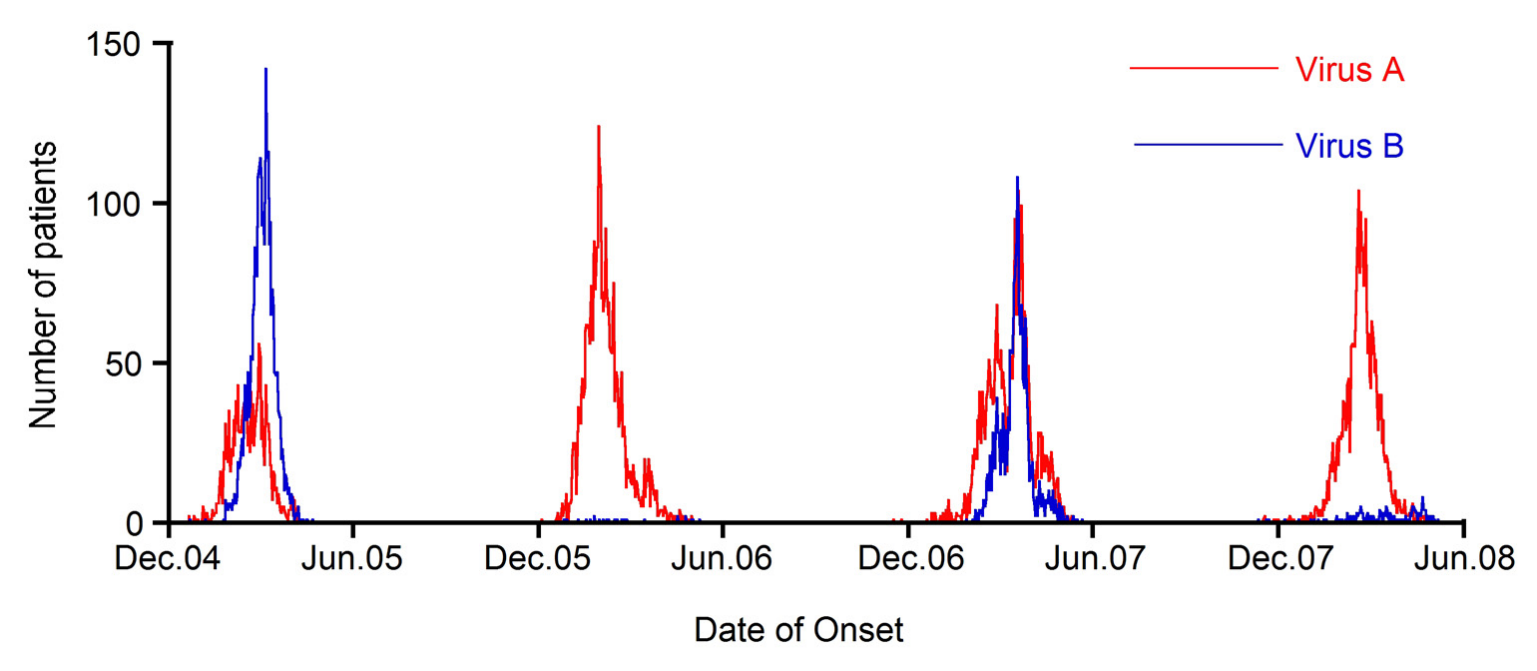
**Epidemic curves for reported patients with influenza A and B virus in 4 seasons**.

The annual influenza season began between November and December, peaked between February and March, and returned to baseline between April and June. Mixed circulation of influenza A and B was confirmed in the 2004/05 and 2006/07 seasons. There were few cases of influenza B in the 2005/06 and 2007/08 seasons (Figure 1).

**Figure 2:**
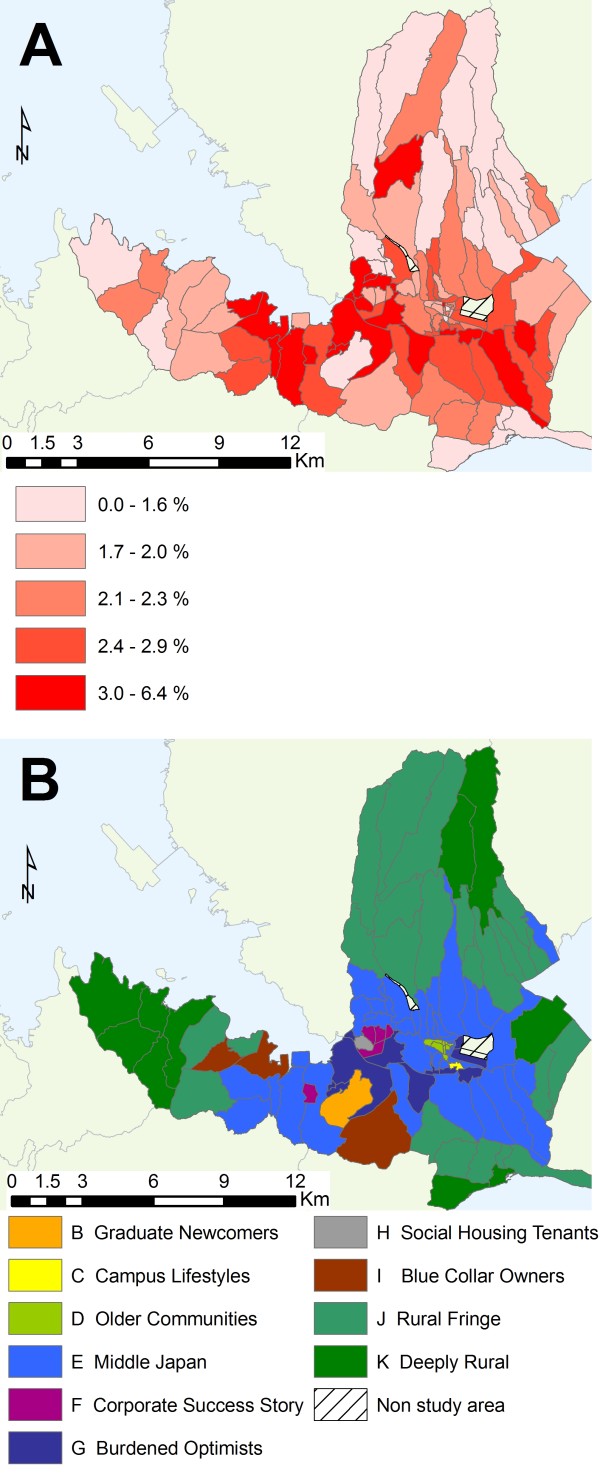
**Relationship between influenza incidence and geodemographics profiling in the study area**. (A). Map of the incidence rate of influenza A in the 2004/05 to 2007/08 seasons in the study area by census enumeration district. (B). Map of geodemographics profiling in the study area by Mosaic Group.

The average influenza incidence for all ages in the study area during the four seasons was 2.5% for influenza A and 1.1% for influenza B (Table 2). Age group analysis showed that the incidence of influenza A and B was higher in the 5-9 year age group than in others in all four seasons, while that of influenza B was higher in the 10-14 year age group than in other age groups in the 2006/07 season. Geographically, the incidence of influenza A during the four seasons by census enumeration district was higher in the center of the city and was lower in the outskirts (Figure 2, panel A). However, disparities in consultation behaviors caused by traveling, such as rural residents seeking consultation in the city area, could not be evaluated because of the lack of information on medical facilities that the patients visited.

**Table 2:** Numbers of influenza A and B patients and their incidence by age group^†^

		2004/05 Season		2005/06 Season		2006/07 Season		2007/08 Season	
		
Age Group (yr)	Population **No**.	No. patients	II (%)	No. patients	II (%)	No. patients	II (%)	No. patients	II (%)
Virus Type A									
< 5	5,553	368	6.6	702	12.6	452	8.1	458	6.6
5-9	5,857	433	7.4	772	13.2	787	13.4	794	7.4
10-14	6,263	271	4.3	462	7.4	581	9.3	466	4.3
15-64	73,365	560	0.8	1,348	1.8	1,441	2.0	1,117	0.8
≥ 65	21,858	54	0.2	97	0.4	103	0.5	53	0.2
Total	112,901	1,686	1.5	3,381	3.0	3,364	3.0	2,888	1.5

Virus Type B									
< 5	5,553	569	10.2	2	0.0	165	3.0	40	0.7
5-9	5,857	743	12.7	3	0.1	569	9.7	70	1.2
10-14	6,263	219	3.5	4	0.1	803	12.8	18	0.3
15-64	73,365	1,287	1.8	16	0.0	436	0.6	45	0.1
≥ 65	21,858	142	0.6	0	0.0	11	0.1	4	0.0
Total	112,901	2,960	2.6	25	0.0	1,984	1.8	177	0.2

^† ^Population No., Number of people in the study area. No.patients, Number of patients in the study area each season.

II, Influenza incidence in the study area.

### Evaluation of Incidence of Influenza by Geoprofiling (non-age-adjusted and age-adjusted conditions)

There were 10 Mosaic Groups (B to K) and 24 Mosaic Types in the study area. The "Middle Japan" and "Burdened Optimists" groups were mainly distributed in the center of the study area (Figure 2, panel B).

The IVII values for influenza A in the "Burdened Optimists" group were over 100 (range 117-142); the observed numbers were higher than those expected, with the difference being statistically significance (p < 0.001, chi-square test) for all four seasons. The IVII values were over 100 for the "Social Housing Tenants" and "Blue Collar Owners" groups; the observed numbers were significantly higher than those expected in two seasons (Table 3).

**Table 3:** Incidence of Influenza A and B patients by Mosaic Japan Groups (Non-age-adjusted condition) ^†^

Geodemographics Profile			Incidence of Influenza A							
**Mosaic Group** **Description**	**Population** **No.**	**Population** **Density**	**2004/05** **Season**		**2005/06** **Season**		**2006/07** **Season**		**2007/08** **Season**	
			
			**No.**	**IVII**	**No.**	**IVII**	**No.**	**IVII**	**No.**	**IVII**

B: Graduate Newcomers	15	5	0	0	0	0	0	0	0	0
C: Campus Lifestyles	262	1,914	7	179	5	64	7	90	9	134
D: Older Communities	1,200	2,466	13	73	33	92	18	50**	25	81
E: Middle Japan	57,443	1,158	877	102	1,670	97	1,689	99	1,476	100
F: Corporate Success Story	8,121	5,261	97	80*	248	102	249	103	199	96
G: Burdened Optimists	19,781	2,323	356	121***	840	142***	690	117***	691	137
H: Social Housing Tenants	3,837	10,167	56	98	151	131***	156	136***	77	78
I: Blue Collar Owners	2,759	289	96	233***	81	98	111	135**	84	119
J: Rural Fringe	13,495	188	138	68***	272	67***	300	75***	239	69
K: Deeply Rural	5,988	161	46	51***	81	45***	144	81**	88	57

**Geodemographics Profile**			**Incidence of Influenza B**							

**Mosaic Group** **Description**	**Population** **No.**	**Population** **Density**	**2004/05** **Season**		**2005/06** **Season**		**2006/07** **Season**		**2007/08** **Season**	
			
			**No.**	**IVII**	**No.**	**IVII**	**No.**	**IVII**	**No.**	**IVII**

B: Graduate Newcomers	15	5	0	0	-	-	0	0	-	-
C: Campus Lifestyles	262	1,914	8	116	-	-	2	43	-	-
D: Older Communities	1,200	2,466	27	86	-	-	9	43	-	-
E: Middle Japan	57,443	1,158	1,555	103	-	-	1,031	102	-	-
F: Corporate Success Story	8,121	5,261	181	85	-	-	93	65	-	-
G: Burdened Optimists	19,781	2,323	593	114	-	-	451	130	-	-
H: Social Housing Tenants	3,837	10,167	118	117	-	-	39	58	-	-
I: Blue Collar Owners	2,759	289	107	148	-	-	52	107	-	-
J: Rural Fringe	13,495	188	277	78	-	-	194	82	-	-
K: Deeply Rural	5,988	161	94	60	-	-	113	107	-	-

Population No., Number of people in each Mosaic Group. Population Density, Number of people per 1 square kilometer in each Mosaic Group.

No., Number of patients by Mosaic Group each season. IVII, Index value of influenza incidence by Mosaic Group each season.

IVII of Influenza B in the 2005/06 and 2007/08 seasons weren't calculated because of few cases.

* p < 0.05, ** p < 0.01, *** p < 0.001

In contrast, the IVII values for influenza A were under 100 (range 51-81) for the "Rural Fringe" and "Deeply Rural" groups; the observed numbers were significantly lower than those expected in all four seasons (p < 0.001). The IVII values were under 100 for the "Older Communities", "Corporate Success Story" and "Social Housing Tenants" groups; the observed numbers were significantly lower than those expected in one season (Table 3).

After age adjustment, the number of influenza A patients in the "Burdened Optimists" group in the 2005/06 and 2007/08 seasons (p < 0.001) and "Blue Collar Owners" group in the 2004/2005 and 2006/2007 seasons remained statistically significant with higher than expected values in these seasons (Table 4). On the other hand, the index values for the "Rural Fringe" and "Deeply Rural" groups remained significantly lower than those expected in all seasons, with an exception being the "Deeply Rural" group, which did not have a significantly different value in the 2006/2007 season. The significance for the lower index values of the "Social Housing Tenants" and "Blue Collar Owners" groups remained unchanged.

**Table 4:** Incidence of Influenza A and B patients by Mosaic Japan Groups (Age-adjusted condition) ^†^

Geodemographics Profile			Incidence of Influenza A							
**Mosaic Group** **Description**	**Population** **No.**	**Population** **Density**	**2004/05** **Season**		**2005/06** **Season**		**2006/07** **Season**		**2007/08** **Season**	
			
			**No.**	**IVII**	**No.**	**IVII**	**No.**	**IVII**	**No.**	**IVII**

B: Graduate Newcomers	15	5	0	0	0	0	0	0	0	0
C: Campus Lifestyles	262	1,914	7	172	5	61	7	86	9	128
D: Older Communities	1,200	2,466	13	103	33	127	18	67	25	114
E: Middle Japan	57,443	1,158	877	104	1,670	98	1,689	100	1,476	102
F: Corporate Success Story	8,121	5,261	97	86	248	109	249	109	199	103
G: Burdened Optimists	19,781	2,323	356	102	840	121***	690	102	691	116***
H: Social Housing Tenants	3,837	10,167	56	77	151	106	156	111	77	62***
I: Blue Collar Owners	2,759	289	96	209**	81	89	111	123	84	107
J: Rural Fringe	13,495	188	138	79**	272	77***	300	83***	239	78***
K: Deeply Rural	5,988	161	46	66**	81	58***	144	100	88	73***

**Geodemographics Profile**			**Incidence of Influenza B**							

**Mosaic Group** **Description**	**Population** **No.**	**Population** **Density**	**2004/05** **Season**		**2005/06** **Season**		**2006/07** **Season**		**2007/08** **Season**	
			
			**No.**	**IVII**	**No.**	**IVII**	**No.**	**IVII**	**No.**	**IVII**

B: Graduate Newcomers	15	5	0	0	-	-	0	0	-	-
C: Campus Lifestyles	262	1,914	8	110	-	-	2	44	-	-
D: Older Communities	1,200	2,466	27	115	-	-	9	63	-	-
E: Middle Japan	57,443	1,158	1,555	104	-	-	1,031	107	-	-
F: Corporate Success Story	8,121	5,261	181	91	-	-	93	68	-	-
G: Burdened Optimists	19,781	2,323	593	99	-	-	451	109	-	-
H: Social Housing Tenants	3,837	10,167	118	97	-	-	39	43	-	-
I: Blue Collar Owners	2,759	289	107	137	-	-	52	91	-	-
J: Rural Fringe	13,495	188	277	89	-	-	194	88	-	-
K: Deeply Rural	5,988	161	94	74	-	-	113	135	-	-

^† ^Population No., Number of people in each Mosaic Group. Population Density, Number of people per 1 square kilometer in each Mosaic Group.

No., Number of patients by Mosaic Group each season. IVII, Index value of influenza incidence by Mosaic Group each season.

IVII of Influenza B in the 2005/06 and 2007/08 seasons weren't calculated because of few cases.

* p < 0.05, ** p < 0.01, *** p < 0.001

In terms of Mosaic Type (the subcategory of the Mosaic Group) without age adjustment, it was notable that the number for influenza A patients in the "Company Towns" type, was significantly higher than that expected in all four seasons (p < 0.001), and the IVII values of this group were the highest among all Mosaic Types (range 199-330). The IVII values were over 100 for the "Small Town Periphery", "Corporative Careerists", "Blue Collar Families", "Small Town Strugglers", "Welfare Dependency" and "New Collective Housing" types; the observed numbers were significantly higher than those expected in more than two seasons (Additional file 1, Table S1).

On the other hand, the IVII value for influenza A was under 100 with statistical significance in more than two seasons in the following Mosaic Types; the "Small Service Centers", "Small Town Seniors", "Lowland Rural Fringe", "Rural Rejuvenation", "Senior Citizen Houses", "Rural Traditions" and "Coast and Mountain" (Additional file 1, Table S1).

After age adjustment for Mosaic Type with influenza A, the number of IVII values over or under 100 with statistical significance was less than that for the non-age adjusted results, but the general tendency of index values being higher or lower than those expected remained consistent (Additional file 1, Table S2).

For influenza B, the number of patients in the "Burdened Optimists" group was significantly higher than that expected in the two analyzed seasons, 2004/2005 and 2006/2007 (p < 0.01), and the IVII values were 114 and 130, respectively, without age adjustment (Table 3). The IVII values were over 100 for the "Blue Collar Owners" group; the observed numbers were significantly higher than those expected in one season (Table 3). On the other hand, the reported numbers of influenza B patients in the "Corporate Success Story", "Rural Fringe", and "Deeply Rural" groups were significantly lower than those expected in all seasons, and their IVII values were lower than 100 (range 60-82). However, this difference in the reported number was not applicable for the "Deeply Rural" group in the 2006/07 season. The IVII values were under 100 for the "Older Communities" and "Social Housing Tenants" groups, and the observed number was significantly lower than that expected in one season (Table 3).

After age adjustment, the numbers of influenza B patients in the "Middle Japan", "Blue Collar Owners" and "Deeply Rural" groups were significantly higher than those expected in one season, but that of the "Burdened Optimists" group lost statistical significance in both seasons (Table 4). The higher index value for the "Deeply Rural" group was attributed to an influenza B outbreak in the long-term care facility for disabled adults. The lower values for other groups in the non-adjustment data, the "Corporate Success Story", "Social Housing Tenants", and "Deeply Rural" groups, remained significant at least in one season after adjustment.

The number of influenza B patients by Mosaic Type in the "Company Towns" was significantly higher than that expected in the 2004/05 season (p < 0.001), but the difference was not statistically significant in the 2006/2007 season in non-adjusted data. The IVII values were over 100 for the "Small Service Centres", "Micro Communities", "Corporative Careerists", "Blue Collar Families", "Small Town Strugglers", "New Collective Housing" and "Rural Traditions" types; the observed numbers were significantly higher than those expected in more than one season (Additional file 1, Table S1).

On the other hand, the index values were significantly lower than expected in the following Mosaic Types in at least one season: "Suburban Elite", "Welfare Dependency", "Small Town Seniors", "Senior Citizen Houses", and "Non Farm Rural Areas" (Additional file 1, Table S1).

### Exploring Age Distribution of Segmented Neighborhood

Next, we explored the age distribution in each Mosaic Group and Type calculated from the census data to elucidate factors related to the higher or lower influenza incidence in the area profiles. The "Middle Japan" group was typical of neighborhood groups in terms of the family structure and socio-economic states of residents in Japan, and the IVAG values for each 5-year age-group were around 100 (range 86-108). The "Burdened Optimists" group was characterized by families consisting of young parents and the IVAG values for the under 15 yr and 25-44 yr age-groups were all over 120. Conversely, the IVAG values for the over 65 yr age-groups were all under 70 (Additional file 2). This tendency was particularly strong in the "Company Town" Mosaic Type G28, and the IVAG values for the 5-9 yr and 35-39 yr age-group were 249 and 224, respectively (Additional file 2).

"Rural Fringe" and "Deeply Rural" groups were characteristic of agricultural areas with an aging society in the peripheral area, and the IVAG values for the over 65 yr age-groups were over 120, while the values for the 0-9 yr and 20-44 yr age-groups were under 100 (Additional file 2).

### Population Density of Segmented Neighborhood

The top three Mosaic Groups in terms of population density were the "Social Housing Tenants", "Corporate Success Story", and "Older Communities" groups, and their values were 10,167; 5,261; and 2,466 persons per km^2^, respectively (Table 3 and Table 4). By contrast, the groups with the lowest three population densities were the "Graduate Newcomers", "Deeply Rural", and "Rural Fringe" groups, and their values were 15; 161; and 188 persons per km^2^, respectively (Table 3 and Table 4), however, the population density of the "Graduate Newcomers" group was too small to draw any conclusions.

Regarding Mosaic Type, the top three types in terms of population density were the "Welfare Dependency", "Company Towns" and "Suburban Elite" types, and their values were 10,167; 6,412; and 5,598 persons per km^2^, respectively (Additional file 1, Table S1 and Table S2). By contrast, if the "Factory Accommodation" type is excluded, the types with the lowest three population densities were the "Rural Traditions", "Factory Towns", and "Small Town Seniors" types, and their values were 15, 161; and 188 persons per km^2^(Additional file 1, Table S1 and Table S2).

## Discussion

To the best of our knowledge, this is the first study to correlate influenza occurrence in a local community with geodemographics data. We found that the incidence of influenza A and B in the neighborhood group "Burdened Optimists" (Mosaic Group G) was 10-40% higher than expected in the study area (Table 3). This group consists of parents in their 30s and 40s living with their children (Table 1 and Additional file 2). Supporting this finding, the "Company Town" (Mosaic Type G28) the subcategory of this group, where many families in their 30s to mid 40s live with children aged 0-14 year (Additional file 2), was approximately 100-230% higher than expected (Additional file 1, Table S1). On the contrary, the incidence of influenza A and B in neighborhood groups with an aging society in rural areas where the proportion of elderly citizens was high (Additional file 2), the "Rural Fringe" and "Deeply Rural" groups (Mosaic Groups J and K) was 20-50% lower than that expected, a difference that was statistically significant (Table 3).

This finding was a reflection of the higher incidence in children and lower incidence in the elderly for influenza A and B drawn from age group analysis in the entire area, but the results tended to be similar even after age adjustment. Therefore, it was suggested that the clustering of children in young families was a cause for the higher transmission of influenza. Children in households play a key role in influenza transmission, and we assume that the parents in their 30s and 40s are also relatively susceptible to influenza compared to the elderly due to greater chances of contact with children and a lesser chance of having a history of past infection.

Population density is also another factor that affects influenza transmission in neighborhoods. Influenza incidence tended to be higher in the "Social Housing Tenants" groups that had the highest population density and with many small children, and the incidence was lower in the sparsely populated neighborhood groups with many elderly, the "Rural Fringe" and "Deeply Rural" groups. However, the group with the fourth highest population density and a high proportion of children, "Burdened Optimists" had a higher influenza incidence during our study period. Thus, the crowding of people in neighborhoods with many small children could explain the increased levels of influenza in such neighborhoods, just as a sparse population with an aging society can explain the low incidence of influenza; however, population density is not the only factor explaining these differences. We have to consider factors such as social contacts, influenza susceptibility by age group and other socioeconomic factors that can help interpret our study results.

During our study period, influenza circulated in all four seasons, but influenza B caused community outbreaks only in two seasons. The alternating circulation patterns of influenza A and B are among the more prevalent characteristics of influenza [30].

Our age specific incidence analysis demonstrated that children have higher attack rates during typical seasonal influenza outbreaks than adults and the elderly (Table 2). Among them, in the 5-9 years age group, the incidence of influenza A was highest in all four seasons, and that of influenza B was highest in the 2004/2005 season. However, in the 10-14 years age group, the incidence of influenza B was highest in the 2006/07 season. A previous community based survey showed that the highest attack rates were observed in children aged <10 years for influenza A and in those aged 10-19 years for influenza B[30]. Furthermore, our observed attack rates regarding age specific incidence were consistent with the age specific characteristics of influenza.

Several reasons are responsible for a high attack rate in children. First, children are more susceptible to influenza than adults because they are immunologically naive with a lower likelihood of previous infections [31]. Second, young children shed influenza virus for longer periods and in higher titers than adults during illness [32,33]. Third, children have frequent social contacts with their schoolmates [20,21].

Social contact studies suggest that individuals in all age groups tend to mix assortatively; in other words, they mix with people of similar age [16-22], especially in the case of children and adolescents [17,20-22]. Furthermore, these studies show that children mix intimately with their parents, particularly for the 30 to 39 year age group, in which such mixing occurs mainly in their homes [20,22]. Simulation studies using data on social contact indicated that school-aged children have the highest incidence of infection and play a major role in the further spread of infection during initial phases of epidemics by respiratory dissemination [17,20]. By using survey-based contact data and mortality data, optimal vaccination is achieved by prioritization of schoolchildren and adults aged 30-39 years [34]. These observations suggest that the virological characteristics of children and their social contacts strongly contribute to influenza transmission in the community.

On the contrary, the "Rural Fringe" and "Deeply Rural" groups, in which the percentages of people in younger age groups were low but those of people in older age groups were high, and the incidence of influenza A and B was a significantly low. Residents in these neighborhoods are mainly engaged in self-employed farming or fishery work. Therefore, infrequent social contact within these neighborhoods, especially the contact of elderly people with virus-carrying children, would result in a relatively low risk for influenza transmission in addition to immunity from past infections [35].

Geodemographics classifies residential areas according to various characteristics, providing geographers with new analytical information to help identify what type of residents live in a neighborhood [28]. These data have been used to study issues related to the social structure and physical environment in small neighborhoods, identified by their zip code or census tract code. In recent years, social marketing principles and techniques have been central to government proposals for improving health and tackling inequalities in health [36]. Geodemographics is used not only in commerce but also in various areas of public heath, such as drug abuse [37], smoking cessation programs [38], Type 2 diabetes [39], primary dental care service [40], and self-rated health [41]. The use of geodemographics profiles offers the possibility of improving our understanding of the probability of the incidence or inequality in them between districts and within communities. The use of this approach enables the health sector to target interventions effectively in some neighborhood groups [36]. In this study, we used a commercially available dataset, Mosaic Japan. A range of geodemographics tools are currently in use, but the ways in which they are constructed are broadly similar. The tools tend to use variables drawn either entirely or in part from the census data. Regarding the Mosaic Japan dataset, a large number of variables were collected from census data and commercial data. Census data including age group, sex, occupational type, working situation, housing type, population density, and other variables were obtained from a commercial database to infer income levels, life styles and consumer behaviors. Many variables were collected at the household level by census research or consumer survey, and they were aggregated at the census enumeration district level. Segmentations were generated by clustering those multi-variables using a multivariate classification method such as K-means cluster analysis [28]. In the case of Mosaic Japan's geodemographics clusters, all 0.2 million Japanese census tracts were classified into 50 different neighborhood types that were then aggregated into 11 neighborhood groups. One of the reasons why we used a commercial database was that Mosaic Japan contains variables not included in the Japanese Census data, such as income level and life style. These variables can potentially influence the profiles of neighbors, but they are difficult to obtain unless expensive surveys are conducted. The advantage of using existing datasets is especially applicable to decision makers, because of the ease in elucidating some of the information inherent in multivariate classification analysis, and eventually one is able to extrapolate results from small areas to wider regions such as prefectures or to the nationwide level if similar profiles exist. Besides, social and economic structures differ from society to society, and census data collections also differ from that among counties. Consequently, each country tends to have its own geodemographics profiling dataset, but these commercial datasets have a universal method of application. This indicates that the existing datasets not only permit interpolation of the results to other areas in Japan but also have a potential application for comparison with datasets of other countries.

It is common in epidemiological studies to list only adjusted results as in the case of standard mortality rate (SMR) such as cancer to evaluate the disease incidence (or mortality) by census enumeration district, municipality, or prefecture. In those epidemiological studies, age distribution is considered a strong factor that affects disease incidence, and age-adjusted calculation is applied to compare regional differences.

However, many of previous geodemographical studies provided non-adjusted results and did not implement adjustment [37,39,42]. In our paper, non-age adjusted results showed that the influenza incidence was high in the segments with young families with children, who had the highest incidence of influenza among age groups, and low where elderly, who had the lowest incidence, dwell, and the age adjusted results demonstrated that the infection rates across generations were still high in the former segments and low in the latter.

Both non-age adjusted and age-adjusted results are valuable for understanding the different effects on the incidence of influenza between the compositional effects of age groups of residents and contextual effects in the community.

Thus, we believe that our findings on influenza may lead to generalized ways of capturing characteristics of influenza circulation in societies. This will particularly be useful for allocation vaccines and anti-influenza drugs to high risk neighborhoods if the number of cases is rapidly growing and the decision maker has to choose target areas with the little delay.

This study has several limitations. Regarding data collection, patient medical consultation seeking behaviors between or among different age groups regarding influenza-like illnesses remained unknown. However, one OECD study showed that the rate for outpatient visits per person in Japan was the highest among all studied countries in 2007 [43]; therefore, non-inclusion of cases because of failure to seek medical attention may be lower than that in other countries. When we compared school absenteeism in elementary and junior high schools in a different season of 2008/09 in Isahaya City, our patient number was twice as high as that for school absenteeism (data not shown). It often happens that the networks of parents and children are strong conduits via which information and decisions are spread. If, for example, one school concludes that it has a concerning number of influenza cases, the children and adults associated with that neighborhood might be on higher alert. They may be quicker to seek medical care and prescription of anti-influenza drugs. This information supported the high consultation rate for influenza-like illness in children, but the other age groups remain uninvestigated. In addition, medical consultation seeking behaviors may be different based on the location of residence. Patients in rural areas may not seek medical service because of difficulties in accessing these services. To our knowledge, no study has been conducted in Japan on the medical consultation rate of patients with influenza-like illnesses in the community. Thus, these problems should be solved by future studies combining the data obtained from social questionnaire surveys and data already in our possession. The influence of selection bias from refusal for registration appeared to be minimal because the Isahaya Medical Association assured that an extremely low number of patients refused to participate in the study; however, the possibility of a larger bias remains after excluding clinically diagnosed and migrated patients who were referred to medical facilities outside the study area.

In the present study, influenza A had consistent results for higher or lower index values for particular Mosaic Groups and Types over the seasons even after age adjustment, but the results for influenza B were less consistent. One reason is that influenza B has different transmission patterns, affecting different age groups and group sizes, which led to slightly different area profiles compared to those for influenza A. In addition, as our study was based on an ecological analysis, we believe it is difficult to accurately determine all the reasons why influenza frequently or infrequently occurs in a particular neighborhood together with possible small number problems [42].

## Conclusions

We believe that understanding the incidence of influenza in neighborhood groups is a valuable basis for community strategies to control influenza and that a simple statistical analysis using geodemographics tool is an effective means to aid the understanding of differences in the incidence of influenza among neighborhood groups. Our results are useful for stake holders in finding areas of priority to allocate vaccines and anti-influenza drugs in the case of a sudden increase in the number of influenza patients in a community. We demonstrated that geodemographics is a potentially powerful method for elucidating the correlation between social aspects in small areas and communicable diseases such as influenza. We aim to continue our study to analyze pandemic influenza in 2009 and other communicable diseases in the future.

## Competing interests

The authors declare that they have no competing interests.

## Authors' contributions

YK participated in the study design, performed statistical analysis and drafted the manuscript. RS participated in the study design and helped to draft the manuscript. YT and YO participated in the data collection. TN participated in the study design and advised statistical analysis. YS, AS and TO performed data processing. HS participated in the study design and the interpretation of data. All authors read and approved the final manuscript.

## Pre-publication history

The pre-publication history for this paper can be accessed here:

http://www.biomedcentral.com/1471-2334/11/36/prepub

## Supplementary Material

Additional file 1 **Table S1**. Incidence of Influenza A and B patients by Mosaic Japan Types (Non-adjusted condition) ^† ^Table S2. Incidence of Influenza A and B patients by Mosaic Japan Types (Age-adjusted condition) ^†^ Click here for file

Additional file 2 **Additional Figures**. Index value of each 5-year age group by Mosaic Group (B to K) and Mosaic Type G28 to illustrate the age structures in each Mosaic Group and Mosaic Type in the study area. Click here for file
