# Supplementary material for: Geodemographics profiling of influenza A and B virus infections in community neighborhoods in Japan
Source: BMC Infect Dis. 2011 Feb 2;11:36. doi: 10.1186/1471-2334-11-36 (PMC3044666; doi:10.1186/1471-2334-11-36)
Supplement: Additional file 1 — Table S1. Incidence of Influenza A and B patients by Mosaic Japan Types (Non-adjusted condition) † Table S2. Incidence of Influenza A and B patients by Mosaic Japan Types (Age-adjusted condition) † [file 1471-2334-11-36-S1.PDF]

**Table S1. Incidence of Influenza A and B patients by Mosaic Japan Types (Non-age-adjusted condition) †**

| Geodemographics Profile | | | Incidence of Influenza A | | | | | | | |
|---|---|---|---|---|---|---|---|---|---|---|
| Mosaic Type | Population | Population | 2004/05 Season | | 2005/06 Season | | 2006/07 Season | | 2007/08 Season | |
| Description | No. | Density | No. | IVII | No. | IVII | No. | IVII | No. | IVII |
| B08: Factory Accommodation | 15 | 5 | 0 | 0 | 0 | 0 | 0 | 0 | 0 | 0 |
| C12: Town Gown Transition | 262 | 1,914 | 7 | 179 | 5 | 64 | 7 | 90 | 9 | 134 |
| D13: Nagaya Housing | 795 | 2,678 | 10 | 84 | 19 | 80 | 12 | 51$^{*}$ | 18 | 89 |
| D15: Second Tier Downtown | 405 | 2,135 | 3 | 50 | 14 | 115 | 6 | 50 | 7 | 68 |
| E18: Small Service Centres | 8,927 | 4,607 | 101 | 76$^{**}$ | 299 | 112 | 173 | 65$^{***}$ | 215 | 94 |
| E20: Micro Communities | 3,604 | 408 | 51 | 95 | 93 | 86 | 101 | 94 | 100 | 108 |
| E21: Small Town Periphery | 29,026 | 940 | 526 | 121$^{***}$ | 808 | 93$^{*}$ | 974 | 113$^{***}$ | 793 | 107 |
| E23: Provincial Renters | 15,886 | 1,988 | 199 | 84$^{*}$ | 470 | 99 | 441 | 93 | 368 | 91 |
| F24: Suburban Elite | 6,283 | 5,598 | 50 | 53$^{***}$ | 169 | 90 | 185 | 99 | 127 | 79$^{**}$ |
| F27: Corporative Careerists | 1,838 | 4,362 | 47 | 171$^{***}$ | 79 | 144$^{***}$ | 64 | 117 | 72 | 153$^{***}$ |
| G28: Company Towns | 1,260 | 6,412 | 62 | 330$^{***}$ | 75 | 199$^{***}$ | 92 | 245$^{***}$ | 77 | 239$^{***}$ |
| G29: Blue Collar Families | 1,088 | 3,778 | 16 | 98 | 63 | 193$^{***}$ | 40 | 123 | 43 | 155$^{**}$ |
| G30: Small Town Strugglers | 17,433 | 2,170 | 278 | 107 | 702 | 134$^{***}$ | 558 | 107 | 571 | 128$^{***}$ |
| H33: Welfare Dependency | 3,837 | 10,167 | 56 | 98 | 151 | 131$^{***}$ | 156 | 136$^{***}$ | 77 | 78$^{*}$ |
| I38: Factory Towns | 450 | 75 | 6 | 89 | 10 | 74 | 6 | 45$^{*}$ | 10 | 87 |
| I40: New Collective Housing | 2,309 | 649 | 90 | 261$^{***}$ | 71 | 103 | 105 | 153$^{***}$ | 74 | 125 |
| J42: Small Service Centres | 1,635 | 423 | 6 | 25$^{***}$ | 31 | 63$^{*}$ | 25 | 51$^{***}$ | 31 | 74 |
| J43: Small Town Seniors | 4,071 | 157 | 54 | 89 | 59 | 48$^{***}$ | 89 | 73$^{**}$ | 65 | 62$^{***}$ |
| J44: Lowland Rural Fringe | 3,572 | 166 | 33 | 62$^{**}$ | 89 | 83 | 71 | 67$^{***}$ | 67 | 73$^{*}$ |
| J45: Rural Rejuvenation | 4,217 | 207 | 45 | 71$^{*}$ | 93 | 74$^{**}$ | 115 | 92 | 76 | 70$^{**}$ |
| K46: Senior Citizen Houses | 1,958 | 3,371 | 14 | 48$^{**}$ | 24 | 41$^{***}$ | 48 | 82 | 19 | 38$^{***}$ |
| K47: Non Farm Rural Areas | 1,016 | 227 | 10 | 66 | 25 | 82 | 22 | 73 | 14 | 54$^{*}$ |
| K48: Rural Traditions | 1,953 | 74 | 16 | 55$^{*}$ | 22 | 38$^{***}$ | 48 | 82 | 42 | 84 |
| K49: Coast and Mountain | 1,061 | 189 | 6 | 38$^{*}$ | 10 | 31$^{***}$ | 26 | 82 | 13 | 48$^{**}$ |

† Population No., Number of people in each Mosaic Type. Population Density, Number of people per 1 square kilometer in each Mosaic Type.

No., Number of patients by Mosaic Type each season. IVII, Index value of influenza incidence by Mosaic Type each season.

* $p < 0.05$, ** $p < 0.01$, *** $p < 0.001$

**Table S1 (Continued). Incidence of Influenza A and B patients by Mosaic Japan Types (Non-age-adjusted condition) †**

| Geodemographics Profile | | | Incidence of Influenza B | | | | | | | |
|---|---|---|---|---|---|---|---|---|---|---|
| Mosaic Type | Population | Population | 2004/05 Season | | 2005/06 Season | | 2006/07 Season | | 2007/08 Season | |
| Description | No. | Density | No. | IVII | No. | IVII | No. | IVII | No. | IVII |
| B08: Factory Accommodation | 15 | 5 | 0 | 0 | 0 | – | 0 | 0 | 0 | – |
| C12: Town Gown Transition | 262 | 1,914 | 8 | 116 | 0 | – | 2 | 43 | 1 | – |
| D13: Nagaya Housing | 795 | 2,678 | 19 | 91 | 0 | – | 6 | 43* | 1 | – |
| D15: Second Tier Downtown | 405 | 2,135 | 8 | 75 | 0 | – | 3 | 42 | 0 | – |
| E18: Small Service Centres | 8,927 | 4,607 | 217 | 93 | 0 | – | 188 | 120* | 8 | – |
| E20: Micro Communities | 3,604 | 408 | 110 | 116 | 1 | – | 80 | 126 | 3 | – |
| E21: Small Town Periphery | 29,026 | 940 | 803 | 106 | 9 | – | 484 | 95 | 48 | – |
| E23: Provincial Renters | 15,886 | 1,988 | 425 | 102 | 5 | – | 279 | 100 | 48 | – |
| F24: Suburban Elite | 6,283 | 5,598 | 86 | 52*** | 0 | – | 55 | 50*** | 3 | – |
| F27: Corporative Careerists | 1,838 | 4,362 | 95 | 197*** | 1 | – | 38 | 118 | 4 | – |
| G28: Company Towns | 1,260 | 6,412 | 65 | 197*** | 2 | – | 23 | 104 | 2 | – |
| G29: Blue Collar Families | 1,088 | 3,778 | 51 | 179*** | 0 | – | 17 | 89 | 0 | – |
| G30: Small Town Strugglers | 17,433 | 2,170 | 477 | 104 | 6 | – | 411 | 134*** | 45 | – |
| H33: Welfare Dependency | 3,837 | 10,167 | 118 | 117 | 0 | – | 39 | 58*** | 1 | – |
| I38: Factory Towns | 450 | 75 | 7 | 59 | 0 | – | 3 | 38 | 2 | – |
| I40: New Collective Housing | 2,309 | 649 | 100 | 165*** | 1 | – | 49 | 121 | 1 | – |
| J42: Small Service Centres | 1,635 | 423 | 29 | 68* | 0 | – | 27 | 94 | 0 | – |
| J43: Small Town Seniors | 4,071 | 157 | 79 | 74** | 0 | – | 52 | 73* | 0 | – |
| J44: Lowland Rural Fringe | 3,572 | 166 | 71 | 76* | 0 | – | 56 | 89 | 1 | – |
| J45: Rural Rejuvenation | 4,217 | 207 | 98 | 89 | 0 | – | 59 | 80 | 7 | – |
| K46: Senior Citizen Houses | 1,958 | 3,371 | 26 | 51*** | 0 | – | 17 | 49** | 0 | – |
| K47: Non Farm Rural Areas | 1,016 | 227 | 4 | 15*** | 0 | – | 12 | 67 | 0 | – |
| K48: Rural Traditions | 1,953 | 74 | 43 | 84 | 0 | – | 67 | 195*** | 1 | – |
| K49: Coast and Mountain | 1,061 | 189 | 21 | 75 | 0 | – | 17 | 91 | 1 | – |

† Population No., Number of people in each Mosaic Type. Population Density, Number of people per 1 square kilometer in each Mosaic Type.

No., Number of patients by Mosaic Type each season. IVII, Index value of influenza incidence by Mosaic Type each season.

IVII of Influenza B in the 2005/06 and 2007/08 seasons weren't calculated because of few cases.

* $p < 0.05$, ** $p < 0.01$, *** $p < 0.001$

## Table S2. Incidence of Influenza A and B patients by Mosaic Japan Types (Age-adjusted condition) †

| Geodemographics Profile | | | Incidence of Influenza A | | | | | | | |
|---|---|---|---|---|---|---|---|---|---|---|
| Mosaic Type | Population | Population | 2004/05 Season | | 2005/06 Season | | 2006/07 Season | | 2007/08 Season | |
| Description | No. | Density | No. | IVII | No. | IVII | No. | IVII | No. | IVII |
| B08: Factory Accommodation | 15 | 5 | 0 | 0 | 0 | 0 | 0 | 0 | 0 | 0 |
| C12: Town Gown Transition | 262 | 1,914 | 7 | 172 | 5 | 61 | 7 | 86 | 9 | 128 |
| D13: Nagaya Housing | 795 | 2,678 | 10 | 128 | 19 | 116 | 12 | 71 | 18 | 131 |
| D15: Second Tier Downtown | 405 | 2,135 | 3 | 63 | 14 | 145 | 6 | 60 | 7 | 85 |
| E18: Small Service Centres | 8,927 | 4,607 | 101 | 78* | 299 | 116*** | 173 | 67*** | 215 | 98 |
| E20: Micro Communities | 3,604 | 408 | 51 | 97 | 93 | 89 | 101 | 96 | 100 | 111 |
| E21: Small Town Periphery | 29,026 | 940 | 526 | 121*** | 808 | 92** | 974 | 113*** | 793 | 107 |
| E23: Provincial Renters | 15,886 | 1,988 | 199 | 87* | 470 | 101 | 441 | 96 | 368 | 94 |
| F24: Suburban Elite | 6,283 | 5,598 | 50 | 64** | 169 | 108 | 185 | 117* | 127 | 95 |
| F27: Corporative Careerists | 1,838 | 4,362 | 47 | 133* | 79 | 114 | 64 | 92 | 72 | 119 |
| G28: Company Towns | 1,260 | 6,412 | 62 | 201*** | 75 | 126* | 92 | 160*** | 77 | 148*** |
| G29: Blue Collar Families | 1,088 | 3,778 | 16 | 89 | 63 | 173*** | 40 | 114 | 43 | 141* |
| G30: Small Town Strugglers | 17,433 | 2,170 | 278 | 93 | 702 | 118*** | 558 | 95 | 571 | 112** |
| H33: Welfare Dependency | 3,837 | 10,167 | 56 | 77 | 151 | 106 | 156 | 111 | 77 | 62*** |
| I38: Factory Towns | 450 | 75 | 6 | 114 | 10 | 93 | 6 | 53 | 10 | 107 |
| I40: New Collective Housing | 2,309 | 649 | 90 | 222*** | 71 | 88 | 105 | 133*** | 74 | 107 |
| J42: Small Service Centres | 1,635 | 423 | 6 | 28*** | 31 | 72 | 25 | 57** | 31 | 83 |
| J43: Small Town Seniors | 4,071 | 157 | 54 | 108 | 59 | 58*** | 89 | 86 | 65 | 75* |
| J44: Lowland Rural Fringe | 3,572 | 166 | 33 | 70* | 89 | 94 | 71 | 73** | 67 | 82 |
| J45: Rural Rejuvenation | 4,217 | 207 | 45 | 80 | 93 | 82 | 115 | 99 | 76 | 77* |
| K46: Senior Citizen Houses | 1,958 | 3,371 | 14 | 72 | 24 | 61** | 48 | 119 | 19 | 57* |
| K47: Non Farm Rural Areas | 1,016 | 227 | 10 | 85 | 25 | 107 | 22 | 92 | 14 | 70 |
| K48: Rural Traditions | 1,953 | 74 | 16 | 58* | 22 | 40*** | 48 | 86 | 42 | 89 |
| K49: Coast and Mountain | 1,061 | 189 | 6 | 53 | 10 | 44** | 26 | 111 | 13 | 67 |

† Population No., Number of people in each Mosaic Type. Population Density, Number of people per 1 square kilometer in each Mosaic Type.

No., Number of patients by Mosaic Type each season. IVII, Index value of influenza incidence by Mosaic Type each season.

* $p < 0.05$, ** $p < 0.01$, *** $p < 0.001$

**Table S2 (Continued). Incidence of Influenza A and B patients by Mosaic Japan Types (Non-age-adjusted condition) †**

| Geodemographics Profile | | | Incidence of Influenza B | | | | | | | |
|---|---|---|---|---|---|---|---|---|---|---|
| Mosaic Type | Population | Population | 2004/05 Season | | 2005/06 Season | | 2006/07 Season | | 2007/08 Season | |
| Description | No. | Density | No. | IVII | No. | IVII | No. | IVII | No. | IVII |
| B08: Factory Accommodation | 15 | 5 | 0 | 0 | 0 | – | 0 | 0 | 0 | – |
| C12: Town Gown Transition | 262 | 1,914 | 8 | 110 | 0 | – | 2 | 44 | 1 | – |
| D13: Nagaya Housing | 795 | 2,678 | 19 | 125 | 0 | – | 6 | 72 | 1 | – |
| D15: Second Tier Downtown | 405 | 2,135 | 8 | 96 | 0 | – | 3 | 50 | 0 | – |
| E18: Small Service Centres | 8,927 | 4,607 | 217 | 95 | 0 | – | 188 | 127** | 8 | – |
| E20: Micro Communities | 3,604 | 408 | 110 | 119 | 1 | – | 80 | 129* | 3 | – |
| E21: Small Town Periphery | 29,026 | 940 | 803 | 104 | 9 | – | 484 | 99 | 48 | – |
| E23: Provincial Renters | 15,886 | 1,988 | 425 | 104 | 5 | – | 279 | 107 | 48 | – |
| F24: Suburban Elite | 6,283 | 5,598 | 86 | 61*** | 0 | – | 55 | 61*** | 3 | – |
| F27: Corporative Careerists | 1,838 | 4,362 | 95 | 163*** | 1 | – | 38 | 82 | 4 | – |
| G28: Company Towns | 1,260 | 6,412 | 65 | 127 | 2 | – | 23 | 61* | 2 | – |
| G29: Blue Collar Families | 1,088 | 3,778 | 51 | 162*** | 0 | – | 17 | 87 | 0 | – |
| G30: Small Town Strugglers | 17,433 | 2,170 | 477 | 93 | 6 | – | 411 | 115** | 45 | – |
| H33: Welfare Dependency | 3,837 | 10,167 | 118 | 97 | 0 | – | 39 | 43*** | 1 | – |
| I38: Factory Towns | 450 | 75 | 7 | 75 | 0 | – | 3 | 42 | 2 | – |
| I40: New Collective Housing | 2,309 | 649 | 100 | 146*** | 1 | – | 49 | 98 | 1 | – |
| J42: Small Service Centres | 1,635 | 423 | 29 | 76 | 0 | – | 27 | 99 | 0 | – |
| J43: Small Town Seniors | 4,071 | 157 | 79 | 88 | 0 | – | 52 | 85 | 0 | – |
| J44: Lowland Rural Fringe | 3,572 | 166 | 71 | 86 | 0 | – | 56 | 92 | 1 | – |
| J45: Rural Rejuvenation | 4,217 | 207 | 98 | 98 | 0 | – | 59 | 83 | 7 | – |
| K46: Senior Citizen Houses | 1,958 | 3,371 | 26 | 71 | 0 | – | 17 | 79 | 0 | – |
| K47: Non Farm Rural Areas | 1,016 | 227 | 4 | 19*** | 0 | – | 12 | 82 | 0 | – |
| K48: Rural Traditions | 1,953 | 74 | 43 | 90 | 0 | – | 67 | 192*** | 1 | – |
| K49: Coast and Mountain | 1,061 | 189 | 21 | 100 | 0 | – | 17 | 133 | 1 | – |

† Population No., Number of people in each Mosaic Type. Population Density, Number of people per 1 square kilometer in each Mosaic Type.

No., Number of patients by Mosaic Type each season. IVII, Index value of influenza incidence by Mosaic Type each season.

IVII of Influenza B in the 2005/06 and 2007/08 seasons weren't calculated because of few cases.

* $p < 0.05$, ** $p < 0.01$, *** $p < 0.001$
