# Supplementary material for: Geodemographics profiling of influenza A and B virus infections in community neighborhoods in Japan
Source: BMC Infect Dis. 2011 Feb 2;11:36. doi: 10.1186/1471-2334-11-36 (PMC3044666; doi:10.1186/1471-2334-11-36)
Supplement: Additional file 2 — Additional Figures. Index value of each 5-year age group by Mosaic Group (B to K) and Mosaic Type G28 to illustrate the age structures in each Mosaic Group and Mosaic Type in the study area. [file 1471-2334-11-36-S2.PDF]

Geodemographics profiling of influenza A and B virus infections in community neighborhoods in Japan

Additional Figures

Index value of each 5-year age group by Mosaic Group (B to K) and Mosaic Type G28 to illustrate the age structures in each Mosaic Group and Mosaic Type in the study area.

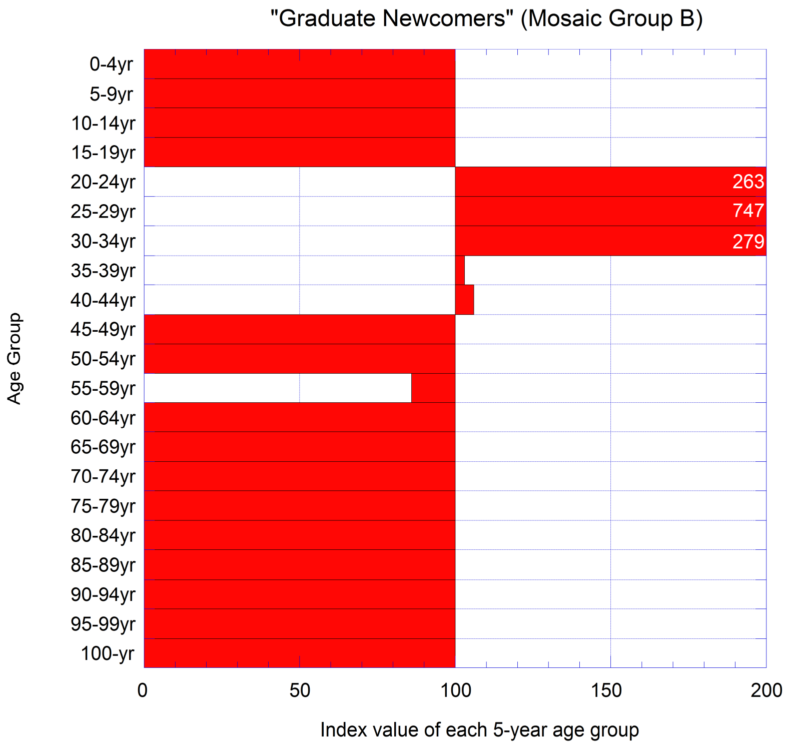

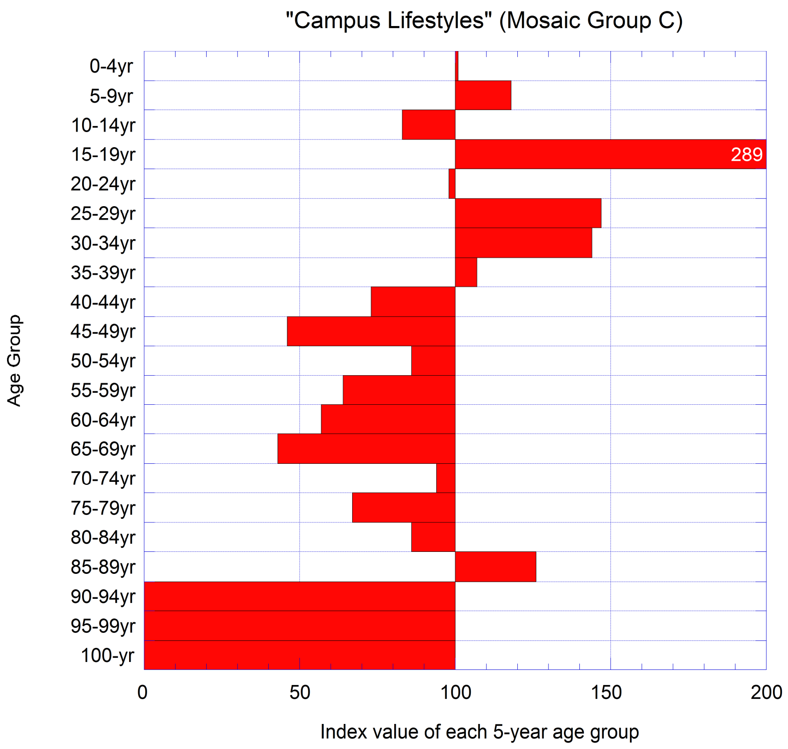
"Campus Lifestyles" (Mosaic Group C)
Age Group
0-4yr
5-9yr
10-14yr
15-19yr
20-24yr
25-29yr
30-34yr
35-39yr
40-44yr
45-49yr
50-54yr
55-59yr
60-64yr
65-69yr
70-74yr
75-79yr
80-84yr
85-89yr
90-94yr
95-99yr
100-yr
289
0
50
100
150
200
Index value of each 5-year age group

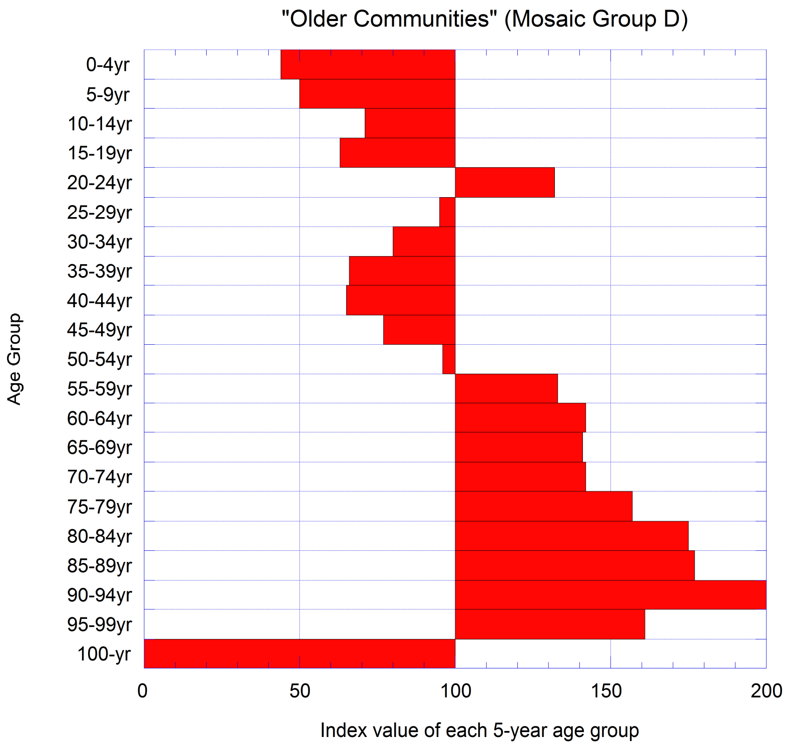
"Older Communities" (Mosaic Group D)
Age Group
0-4yr
5-9yr
10-14yr
15-19yr
20-24yr
25-29yr
30-34yr
35-39yr
40-44yr
45-49yr
50-54yr
55-59yr
60-64yr
65-69yr
70-74yr
75-79yr
80-84yr
85-89yr
90-94yr
95-99yr
100-yr
0
50
100
150
200
Index value of each 5-year age group

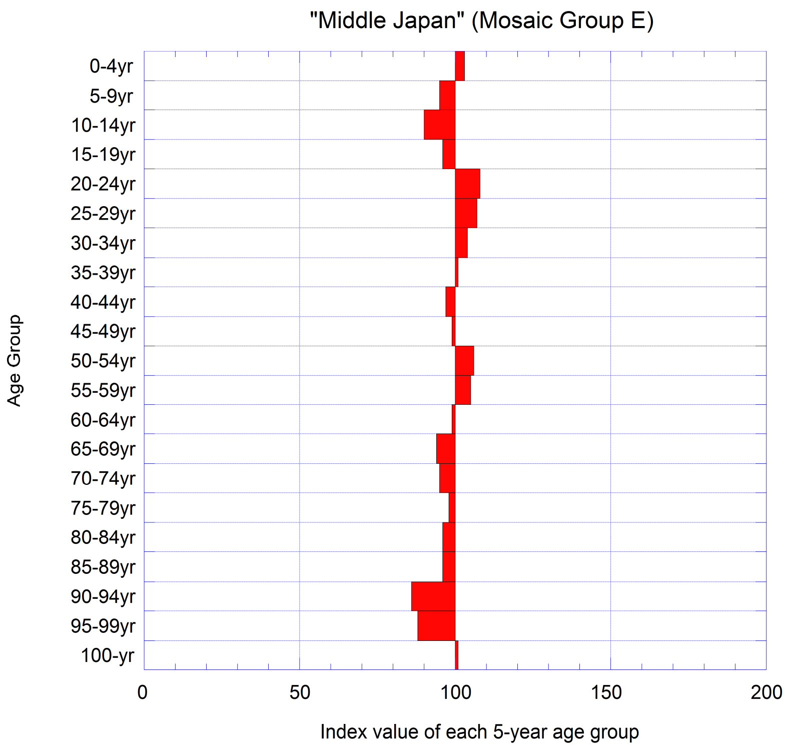
"Middle Japan" (Mosaic Group E)
0-4yr
5-9yr
10-14yr
15-19yr
20-24yr
25-29yr
30-34yr
35-39yr
40-44yr
45-49yr
50-54yr
55-59yr
60-64yr
65-69yr
70-74yr
75-79yr
80-84yr
85-89yr
90-94yr
95-99yr
100-yr
Age Group
0
50
100
150
200
Index value of each 5-year age group

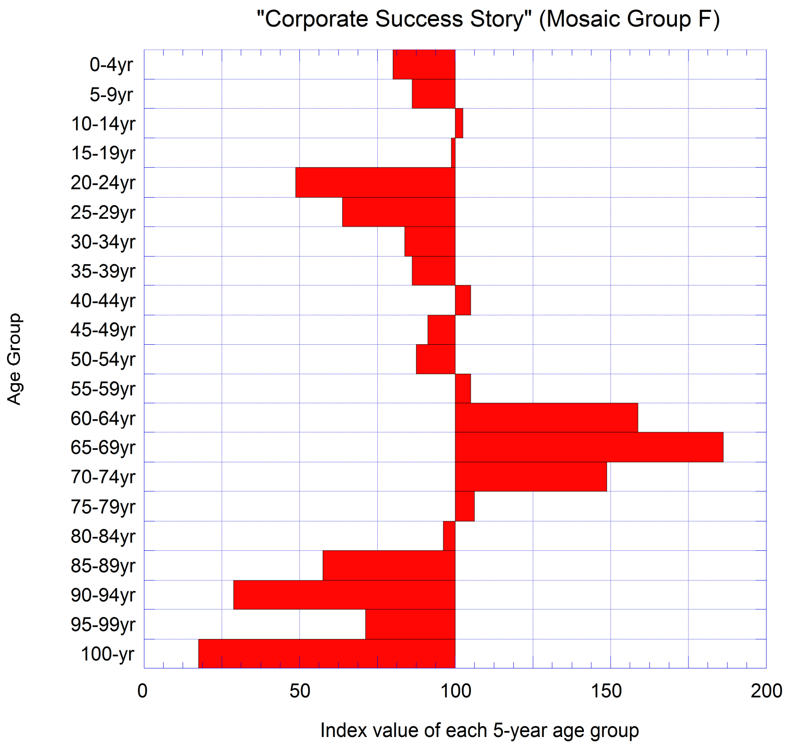
"Corporate Success Story" (Mosaic Group F)
0-4yr
5-9yr
10-14yr
15-19yr
20-24yr
25-29yr
30-34yr
35-39yr
40-44yr
45-49yr
50-54yr
55-59yr
60-64yr
65-69yr
70-74yr
75-79yr
80-84yr
85-89yr
90-94yr
95-99yr
100-yr
Age Group
0
50
100
150
200
Index value of each 5-year age group

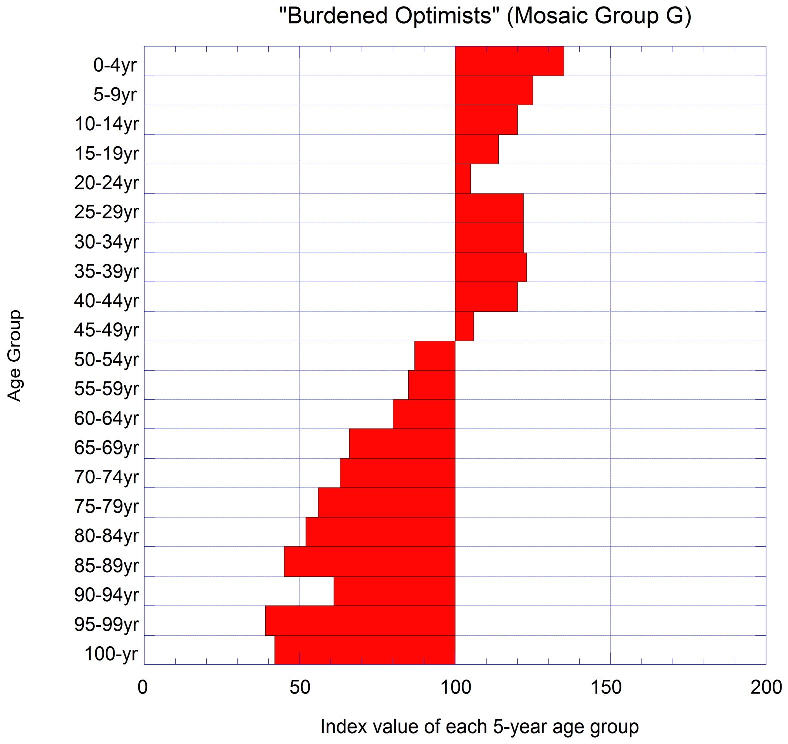
"Burdened Optimists" (Mosaic Group G)
Age Group
0-4yr
5-9yr
10-14yr
15-19yr
20-24yr
25-29yr
30-34yr
35-39yr
40-44yr
45-49yr
50-54yr
55-59yr
60-64yr
65-69yr
70-74yr
75-79yr
80-84yr
85-89yr
90-94yr
95-99yr
100-yr
0
50
100
150
200
Index value of each 5-year age group

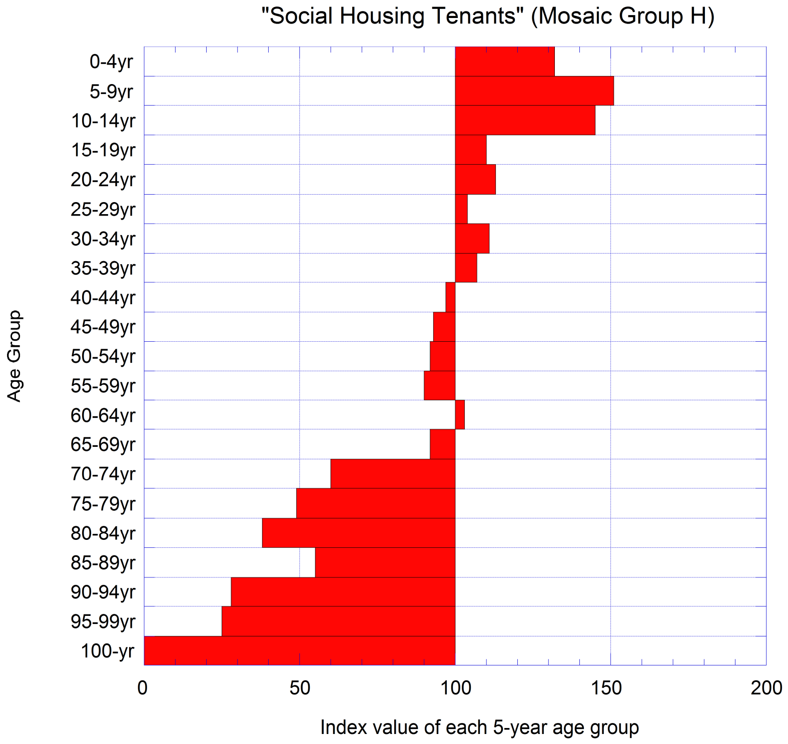
"Social Housing Tenants" (Mosaic Group H)
Age Group
0-4yr
5-9yr
10-14yr
15-19yr
20-24yr
25-29yr
30-34yr
35-39yr
40-44yr
45-49yr
50-54yr
55-59yr
60-64yr
65-69yr
70-74yr
75-79yr
80-84yr
85-89yr
90-94yr
95-99yr
100-yr
0
50
100
150
200
Index value of each 5-year age group

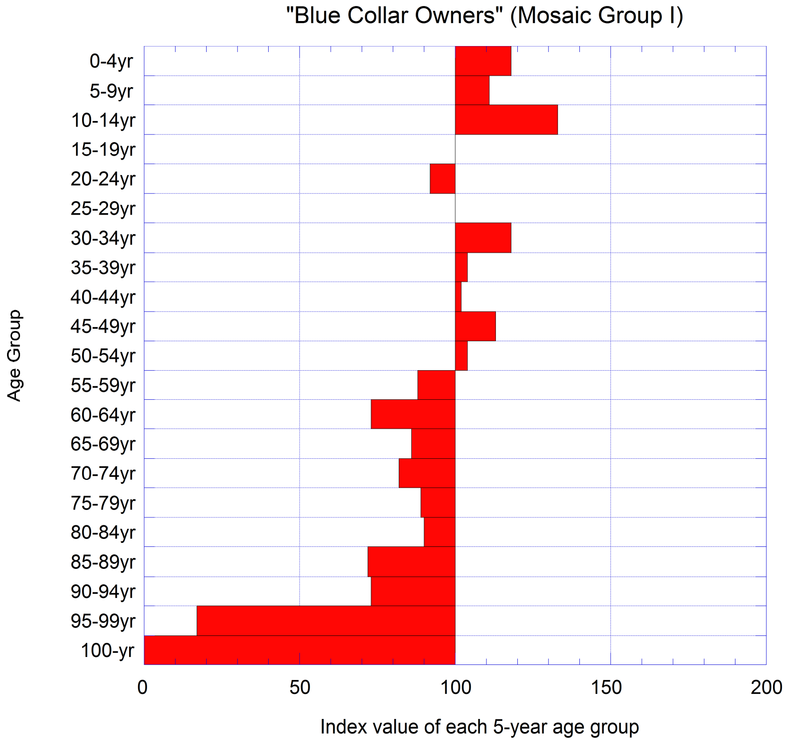
"Blue Collar Owners" (Mosaic Group I)
0-4yr
5-9yr
10-14yr
15-19yr
20-24yr
25-29yr
30-34yr
35-39yr
40-44yr
45-49yr
50-54yr
55-59yr
60-64yr
65-69yr
70-74yr
75-79yr
80-84yr
85-89yr
90-94yr
95-99yr
100-yr
Age Group
0
50
100
150
200
Index value of each 5-year age group

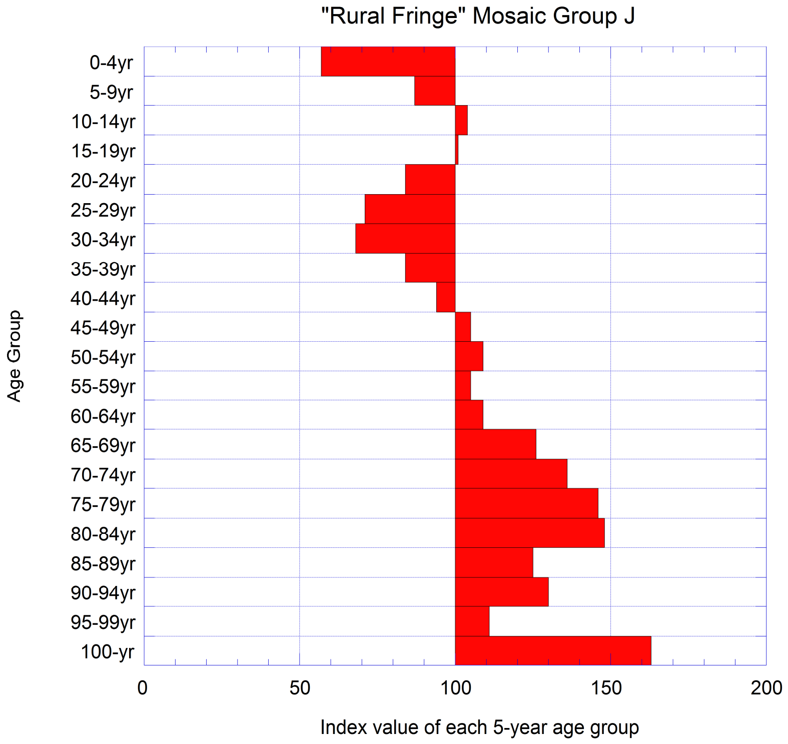
"Rural Fringe" Mosaic Group J
0-4yr
5-9yr
10-14yr
15-19yr
20-24yr
25-29yr
30-34yr
35-39yr
40-44yr
45-49yr
50-54yr
55-59yr
60-64yr
65-69yr
70-74yr
75-79yr
80-84yr
85-89yr
90-94yr
95-99yr
100-yr
Age Group
0
50
100
150
200
Index value of each 5-year age group

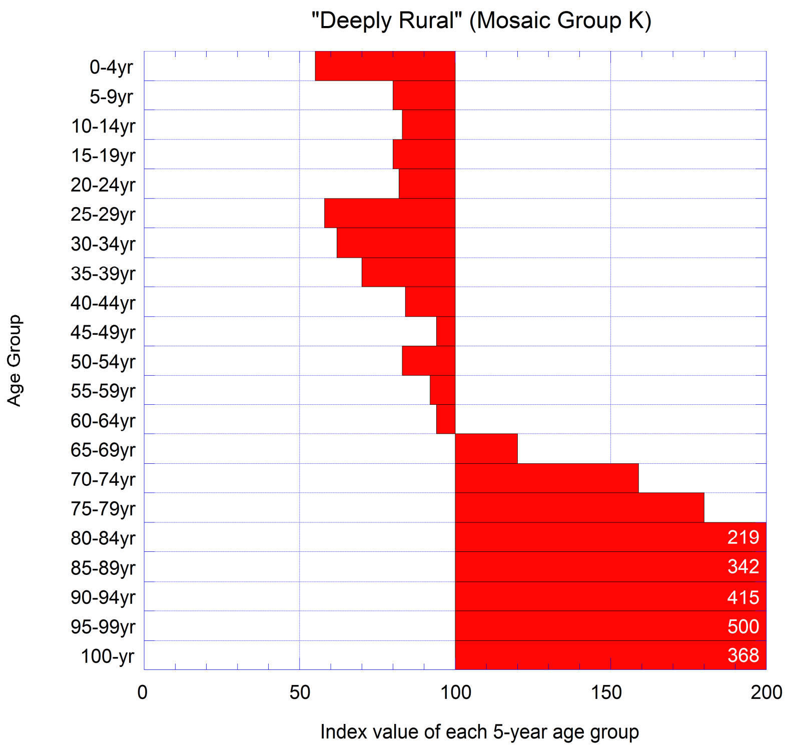
"Deeply Rural" (Mosaic Group K)
Age Group
0-4yr
5-9yr
10-14yr
15-19yr
20-24yr
25-29yr
30-34yr
35-39yr
40-44yr
45-49yr
50-54yr
55-59yr
60-64yr
65-69yr
70-74yr
75-79yr
80-84yr
85-89yr
90-94yr
95-99yr
100-yr
219
342
415
500
368
0
50
100
150
200
Index value of each 5-year age group

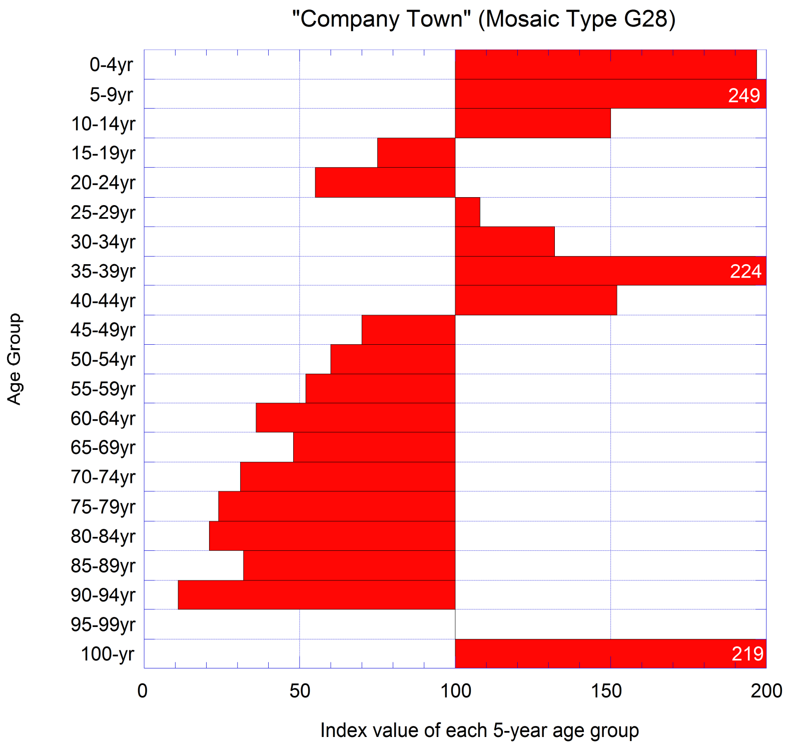
"Company Town" (Mosaic Type G28)
Age Group
0-4yr
5-9yr
10-14yr
15-19yr
20-24yr
25-29yr
30-34yr
35-39yr
40-44yr
45-49yr
50-54yr
55-59yr
60-64yr
65-69yr
70-74yr
75-79yr
80-84yr
85-89yr
90-94yr
95-99yr
100-yr
249
224
219
0
50
100
150
200
Index value of each 5-year age group
